# Exercise stereotypes and health-related outcomes in French people living with HIV: development and validation of an HIV Exercise Stereotypes Scale (HIVESS)

**DOI:** 10.1186/s12955-016-0562-z

**Published:** 2016-11-14

**Authors:** Laura Gray, Charlène Falzon, Alessandro Bergamaschi, Laura Schuft, Jacques Durant, Eric Rosenthal, Christian Pradier, Martin Duracinsky, Isabelle Rouanet, Serge S. Colson, Fabienne d’Arripe-Longueville

**Affiliations:** 1 Université Côte D’azur, LAMHESS, Nice, France; 2 Université Paris-Diderot, EA 7334, (Patient-Centered Outcomes Research), Paris, France; 3 Départment de Maladies Infectieuses, Centre Hospitalier Universitaire de Nîmes, Nimes, France; 4 CHU (Départment de Maladies Infectieuses), Université Côte D’azur, Archet 1, Nice, France; 5 CHU (Département de Santé Publique), Université Côte D’azur, Nice, France; 6 AP-HP, Hopital Bicêtre (Departement de Médecine Interne et d’Immunologie Clinique), Kremlin-Bicetre, France

**Keywords:** HIV/AIDS, Stereotypes, Exercise, Scale development

## Abstract

**Background:**

The main objective of the current study was to develop and validate a French exercise stereotype scale for people living with HIV (PLHIV) in order to gain visibility to the possible barriers and facilitators for exercise in PLHIV and thus enhance their quality of life.

**Methods:**

A series of four complementary studies was carried out with a total sample of 524 participants to: (a) develop a preliminary version of the HIV Exercise Stereotype Scale (HIVESS) (Stage 1), (b) confirm the factorial structure of the instrument (Stage 2), (c) evaluate the stability of the instrument (Stage 3), and (d) examine the construct and divergent validity of the scale (Stage 4).

**Results:**

Results provided support for a 14-item scale with three sub-scales reporting stereotypes related to exercise benefits, exercise risks and lack of capacity for exercise with Cronbach’s alphas of .77, .69 and .76 respectively. Results showed good factorial structure, strong reliability and indicators of convergent validity relating to self-efficacy, exercise and quality of life.

**Conclusion:**

The HIVESS presented satisfactory psychometric properties, constitutes a reliable and valid instrument to measure exercise stereotypes among PLHIV and has applications for future research and clinical practice.

## Background

The positive effects of exercise for people living with HIV (PLHIV) are now thoroughly reported in scientific literature. A recent meta-analysis [1], indicated that moderate exercise presents beneficial physical and psychological effects for this population (e.g., improvement of fitness, body composition and quality of life). Further physical effects noted are the moderation of the side effects of antiretroviral therapy (e.g., reducing and/or preventing uneven distribution of body fat) [2], and of cardiopulmonary fitness [3]. These physical effects coexist with notable psychological benefits such as reduced depression [4, 5] or improved self-perceptions and self-esteem [6]. Greater quality of social relationships [7] and life satisfaction has also been reported comparably to non-exercising PLHIV [8]. Lastly, some studies evidenced the ability of exercise to better antiretroviral therapy adherence [9] and to hinder anti-inflammatory consequences [10].

Regardless of the extensive literature related to the benefits of exercise in PLHIV, a large proportion of this population is not sufficiently active according to current recommendations [11, 12], for adults between 18 and 64 years of age, requiring at least, 150 min of moderate exercise and two sessions of muscle strengthening workout per week [13]. These findings are of concern and encourage further identifying barriers to exercise participation. Research related to exercise barriers in PLHIV is sparse. In a study conducted in Rwandan PLHIV receiving high active antiretroviral therapy [14], the main perceived barriers were lack of motivation (30.5 %), lack of time (25.3 %) and fear of worsening the disease (24.3 %). Through a qualitative study conducted on South African PLHIV, a wide range of obstacles to participation, related to both the psychological sphere (e.g., low-energy levels, psychological complaints and stress levels) and the environmental and social spheres (e.g., physical environment, social environment, domestic abuse and crime) were isolated [15]. More recently, it has been reported that prominent exercise barriers include HIV symptoms (i.e., neuropathy, lipoatrophy), antiretroviral therapy effects (lipodystrophy) and fatigue, but also decreased motivation, depression, negative self-perceptions, and apprehension concerning the risks of exercise such as sustaining an injury [15].

These last results suggest that insufficiently physically active PLHIV might have negative beliefs about exercise effects (e.g., perceived risks), which might limit their participation in exercise. Furthermore, these negative beliefs might reflect the internalization of stereotypes related to physical weakness and decline due to HIV disease. Stereotypes refer to shared beliefs concerning personal characteristics, generally personality traits, and the behaviors of a group of persons [16]*.* According to the stereotype embodiment theory [17], stereotypes are embodied when their assimilation from the surrounding culture leads to self-definitions that, in turn, influence functioning and health. In the case of HIV, internalized stigma has shown a relation to lower mental health and social support, and greater HIV symptoms [18].

An emerging body of literature has focused on the specific influence of exercise stereotypes on different populations such as the elderly or individuals with chronic diseases. Particular measures have been generated to investigate exercise stereotypes in these populations. For example, the “Aging Stereotypes and Exercise Scale” was developed and validated to assess different aspects of aging stereotypes in the exercise sphere such as (a) stereotypes regarding exercise benefits, (b) stereotypes about exercise risks, and (c) stereotypes related to self-efficacy [19]. This line of research indicates that endorsement of negative aging exercise stereotypes is related to lower levels of exercise among the elderly [20, 21]. Another stereotypes-type scale is the “Cancer Exercise Stereotypes Scale” (CESS) [22], which evaluates stereotypes related to exercise in cancer patients: (a) stereotypes related to the lack of interest in exercise; (b) stereotypes regarding exercise self-efficacy; (c) stereotypes about the side effects of treatment; (d) stereotypes related to the risks of exercise and; (e) stereotypes associated with the benefits of exercise. In both scales, some subscales refer to negative exercise stereotypes and others refer to positive exercise stereotypes.

HIV/AIDS-related stigma is continuously brought to mind, in any discussion about effective responses to the epidemic, as a problem of detrimental and tenacious nature [23], several internalized AIDS-stigma scales have hereby been developed [24, 25]. However, no valid tool is currently available to measure stereotypes related to exercise in PLHIV. Taking into consideration the theoretical and conceptual link between exercise stereotypes and behaviors, there is a need to measure stereotypes that act as barriers or facilitators to exercise in PLHIV. This study’s main objective was thus to develop and validate the HIV Exercise Stereotypes Scale (HIVESS). The HIVESS could be used (1) in research to better explain the psychosocial factors related to exercise in PLHIV, and (2) in practice to enable health and exercise professionals to conceive effective intervention strategies that take into account exercise barriers and how to modify them favorably.

## Methods

The HIVESS was developed and validated following Vallerand’s procedure [26], including: (i) developing an initial version and assessing the clarity of the items, (ii) analyzing and confirming the factorial structure of the instrument, (iii) analyzing the stability of the tool over time, and (iv) evidencing proof of construct validity by testing the convergent validity and discriminant validity of the scale.

To perform the successive stages in developing and validating the scale, a total of 420 voluntary French-speaking PLHIV (M_age_ = 52.51; SD = 11.44), 154 voluntary French-speaking healthy individuals (M_age_ = 28.04; SD = 10.45), including 50 French-speaking faculty students (M_age_ = 23.08; SD = 2.25) were recruited Participants were informed of the study during routine consultations or various announcements on social media and in a local newspaper. PLHIV were recruited from three French hospitals by hospital staff and researchers following certain inclusion criteria (i.e., at least 10 years of therapy; no co-infection or other diseases). Healthy individuals and students were recruited in the local area by researchers. Ethical approval as well as participants’ informed written consent was obtained for these studies.

### Stage 1: exploratory version and content clarity

In the first stage of this study the aim was to compile an initial version of the HIVESS. Three researchers specialized in the field of health psychology and four PLHIV formed an ad hoc committee and stipulated a series of items based on (i) content analyses of previous semi-structured interviews focused on exercise beliefs in PLHIV and (ii) two validated scales measuring different dimensions of exercise stereotypes in older adults and cancer patients [19, 22]. Items were thus theoretically prompted and formulated using conventional recommendations for understanding and wording literacy [27]. The concepts of qualitative saturation decided the number of initial items. Saturation was deemed attained when no new relevant item emerged [28]. During this process and in anticipation of items being deleted during the refinement process, items were added to each stereotype category [29]. The Delphi method was then used to select items [30]. The list of items was presented repeatedly until at least 80 % of the raters came to a consensus [31]. A first panel of PLHIV was asked to complete an assessment of the investigative version of the instrument after an initial expert panel review. Participants were invited to assess the clarity of each item on a six-point Likert scale ranging from 1 = “not at all clear” to 6 = “completely clear”. Each participant was interviewed as this qualitative procedure allowed discussing each item as to its pertinence, endorsement and highlighting possible needs for alterations. As suggested by Morey (2003) [29] participants were explicitly encouraged to provide feedback on items rated as being low in quality. The evaluated items, to which mean scores of “4 and/or less” were attributed, were considered as ambiguous or misunderstood. These items where then modified or reformulated according to participant comments and suggestions made by the researchers [26]. A second panel of PLHIV in a method comparable to the first evaluation protocol then assessed the revised version of the instrument.

### Stage 2: factorial structure analysis

This stage aimed at examining the factorial structure of the HIVESS. As item generation was theory driven and based on existing exercise stereotypes scales (e.g., ASES [19]; CESS [22]), the number of factors was predefined. Therefore, maximum likelihood estimation confirmatory factorial analysis (CFA) was conducted in AMOS v7.0 to test the validity of the factor model [32]. Factor analysis is considered possible with a minimal sample of questionnaire items amounting to five times the number of items [33]. Each item was identified as an indicator of a single underlying stereotype sub-scale, and no items were specified to cross-load. The following indicators: chi square (*χ* ^2^; significant values p ≤ .05), the Comparative Fit Index (CFI; values above 0.90), the Tucker-Lewis Index (TLI; values above 0.90), the Root Mean Square Error of Approximation (RMSEA; values below 0.08), and the 90 % Confidence Interval of the RMSEA (RMSEA 90 % CI; values ranging from 0 to 0.08) were used to assess competence of the model fit [34–36]. Sources of poor fit were examined through modification indices [37]. Cronbach’s alphas [38] were also determined to test the internal consistency of each subscale, with convention recommending values greater than 0.70.

### Stage 3: temporal stability

Temporal stability, or “test-retest”, is an important measure of reliability for a psychometric instrument [39]. The purpose of this stage was to assess the temporal accuracy of the HIVESS. An instrument is considered relevant when a reasonable level of temporal stability can be associated to the determined construct measures. For assessment of test-retest reliability at least 50 participants is required for the sample [40].

An interval of 4 weeks was considered to be an acceptable compromise between an attempt to reduce recall bias and test reliability independent of significant clinical change [41]. The 17-item questionnaire was hereby administered to participants twice over a 4-week period. Intraclass correlation coefficients (ICCs) and the 95 % confidence interval of the ICCs (ICC 95 % CI), as well as paired/dependent sample *t*-tests were conducted as data analyses to examine significant differences in subscale scores from time 1 to time 2. Cronbach’s alphas were calculated at both time points to examine internal consistency of the scale.

### Stage 4: convergent validity and discriminant validity

Stage 4 was intended to test the construct validity of the HIVESS. Here we considered the extent to which inferences can be made permissibly in the process of rigorously defining variables into measurable factors. For construct validity the theoretical constructs on which the process was based need to be empirically and qualitatively measured. Construct validity is said to be satisfied when both convergent and discriminant validities are satisfied [42]. A significant correlation of 0.3 is desired between the scale and other theoretically appropriate measures to test validity [43]. Using an alpha of 0.05 and a 1-beta equal to 0.2, 85 participants were needed to obtain a significant correlation of 0.3 or more.

Based on research on exercise stereotypes in vulnerable populations [21, 44], convergent validity of the scale was tested by examining the relationships between the three subscales of the HIVESS and a physical activity score [45], exercise self-efficacy [46] and patient-reported health-related quality of life [47]. Pearson correlation coefficients were used to measure the association between variables. Cronbach’s alphas were examined as internal consistency coefficients.

As past research has reported differences between exercise stereotypes in healthy individuals and people with chronic diseases [22] or older adults [19], we examined discriminant validity by evaluating mean differences on the HIVESS for PLHIV and healthy individuals. Specifically, significant differences were tested using univariate analyses of covariance (ANCOVAs) while controlling for level of exercise, age and gender. Based on the ANCOVA result, the two groups were expected to differ in terms of HIV exercise stereotypes. To assess level of exercise, all participants completed the “Physical Activity Score” [45]. The internal consistency of the items scores was examined using Cronbach’s alpha coefficients.

## Results

### Stage 1: preliminary version and content clarity

Based on previous literature and content analysis of semi-structured interviews with PLHIV, to begin with the ad hoc committee created a pool of 30 items, ten items for each of the three following categories of stereotypes: (i) stereotypes related to exercise benefits, (ii) stereotypes related to risks of exercise, and (iii) stereotypes related to lack of capacity for exercise. The committee followed the Delphi method and retained 5 items for the benefits of exercise subscale and 6 items for the subscales related to the risks of exercise and to lack of capacity for exercise. The examination of the 17 items from the first panel of PLHIV (*N* = 15; M_*age*_ = 50.25; SD = 9.96) reported some low scores for clarity across four items (3.62 < M < 3.93). The evaluation interviews brought to light comments enabling modifications to be made to two items related to risks of exercise and two items related to lack of capacity for exercise. Higher mean clarity scores across all items (M = 5.01; SD = .85) were produced following evaluation of item clarity with the second sample of PLHIV (*N* = 15; M_*age*_ =50.83; SD =10.21) and discussions instigated no further modifications to the items (see Table 1). Stage 1 provided the initial 17-item HIVESS that required psychometric testing. Items were rated on a 6-point Likert scale in order to emphasize the discrimination and reliability and to reduce the risks of deviation linked to personal decision-making [48].

**Table 1:** Items and Clarity Scores for the Preliminary Version of the HIVESS

Dimensions	Items	Clarity Scores (M)
Stereotypes related to benefits of exercise	1. La pratique d’une activité physique permet d’améliorer le moral des patients atteints de VIH^b^ *Exercise improves the morale of HIV-infected patients*	5.6
	2. La pratique d’une activité physique réduit les effets de la maladie chez les patients atteints de VIH^b^ *Exercise reduces the effects of the disease in* *HIV-infected patients*	4.8
	3. La pratique d’une activité physique améliore le bien-être des patients atteints de VIH^b^ *Exercise enhances well-being in HIV-infected patients*	5.6
	4. La pratique d’une activité physique permet aux patients atteints de VIH de se sentir mieux physiquement^b^ *Exercise allows HIV-infected patients to feel better physically*	5.3
	5. La pratique d’une activité physique améliore la survie chez les patients atteints de VIH^b^ *Exercise betters the chances of survival in HIV-infected patients*	4.1
Stereotypes related to risks of exercise	1. Les patients atteints de VIH ne font pas d’activité physique car ils peuvent contaminer quelqu’un au cours de la pratique^b^ *HIV-infected patients do not exercise because they can contaminate someone during the activity*	4.2
	2. La pratique d’une activité physique doit être évitée par les patients atteints de VIH car elle provoque des blessures^b^ *Exercising should be avoided by HIV-infected patients because it causes injuries*	4.1
	3. Les patients atteints de VIH ne font pas d'activité physique car ils redoutent de contaminer quelqu'un au cours de la pratique^b^ *HIV-infected patients do not exercise because they fear contaminating someone during this activity*	4.8
	4. La pratique d’une activité physique est dangereuse pour les patients atteints de VIH car elle entraîne trop d’essoufflements^a^ *Exercising should be avoided by HIV-infected patients because it causes too much breathlessness*	5.1
	5. Les patients atteints de VIH ne font pas d’activité physique car ils peuvent contaminer quelqu’un suite à une blessure^b^ *HIV-infected patients do not exercise because they could contaminate someone after getting injured*	4.9
	6. La pratique d’une activité physique entraîne trop de fatigue chez les patients atteints de VIH^a^ *Exercising causes too much fatigue for HIV-infected patients*	5.5
Stereotypes related to lack of capacity for exercise	1. Les patients atteints de VIH n’ont pas les ressources physiques suffisantes pour faire de l’activité physique^b^ *HIV-infected patients do not have enough physical resources to exercise*	5.1
	2. Les patients atteints de VIH ont des capacités physiques trop limitées pour faire de l'activité physique à cause des traitements^a^ *HIV-infected patients’ physical capacities are too limited to exercise due to their treatments*	5.2
	3. Les patients atteints de VIH ne sont pas capables physiquement de faire de l'activité physique^b^ *HIV-infected patients are not physically apt to exercise*	4.9
	4. A cause des traitements, les patients atteints de VIH n’ont plus assez d’énergie pour faire de l'activité physique^b^ *Because of treatments, HIV-infected patients do not have enough energy to exercise*	5.3
	5. Les douleurs musculaires et articulaires causées par les traitements empêchent les patients atteints de VIH de faire de l'activité physique^b^ *Muscle and joint pain caused by the treatments prevent HIV-infected patients from exercising*	5.2
	6. Les patients atteints de VIH ne font pas d’activité physique car ils sont trop fatigués par les traitements^b^ *HIV-infected patients do not exercise because they are too tired due to their treatments*	5.6

*Notes:* English translations are in italics. For each item, the participant had to answer on a 6-point Likert-type scale from 1 (*do not agree at all*) to 6 (*totally agree*). M = Mean clarity scores

^a^Deleted items following the first CFA

^b^Retained items in the final model

### Stage 2: factorial structure analysis

A total of 96 PLHIV (M_*age*_ = 49.95; SD = 10.61) completed the scale. A second sample of 133 PLHIV (M_*age*_ = 50.46; SD = 10.88) was recruited to complete a modified version of the scale.

The first CFA results did not display a good fit [i.e., χ^2^ (116, *N* = 96) = 267.83; CFI = .74; TLI = .78; RMSEA = .12 (90 % CI = .10, .14)]. Retained items should saturate with a weight greater than .55 as stated by Guttman [49]. Hereby, 3 of the 17 initial items were removed (i.e., two items on the subscale related to exercise risks and one item on the subscale related to lack of capacity for exercise). Using data from the same sample, a second CFA was conducted with the 14-item scale and showed a better fit [i.e., χ^2^ (74, *N* = 96) = 98.99; CFI = .94; TLI = .93; RMSEA = .06 (90 % CI = .02, .09] (see Table 1). The model was then tested with a second sample of 133 PLHIV and also showed a satisfactory fit [i.e., χ^2^ (74, *N* = 133) = 126.51; CFI = .92; TLI = .91; RMSEA = .07 (90 % CI = .05, .09)]. Cronbach’s alphas were .81 for exercise benefits, .71 for exercise risks and .85 for lack of capacity for exercise. Stage 2 rendered and supported the factorial structure of a 14-item, three-factor model of the HIVESS instrument representing HIV exercise stereotype categories related to (i) exercise benefits, (ii) exercise risks, and (iii) lack of capacity for exercise.

### Stage 3: temporal stability

In this stage, 50 faculty students (M_*age*_ = 23.08; SD = 2.25) were recruited to test the temporal stability of the tool. This population was selected as the questionnaire is not only destined to PLHIV as a targeted group, but also to healthy individuals as the targeters. The *t*-tests results are presented in Table 2. No significant differences were found between time 1 and time 2 in the scores within subscales. Cronbach’s alpha coefficients for all three factors at time 1 were .71, .78, .76 for exercise benefits, exercise risks and lack of capacity for exercise respectively. At time 2, Cronbach’s alpha coefficients were .80 for exercise benefits, .82 for exercise risks and .75 for lack of capacity for exercise. Based on these findings, the HIVESS was stable over a 4-week period and internal reliability was persistently demonstrated.

**Table 2:** Descriptive Statistics for the Questionnaire Structure in Stage 3

	Time 1 *(N = 50)*		Time 2 *(N = 50)*		
	M (SD)	α	M (SD)	α	*t*-tests
LCE	3.05 (.81)	.77	3.18 (.78)	.75	*t*(98) = -.83, *p* = .41
RE	2.50 (1.09)	.78	2.42 (1.12)	.82	*t*(98) = .34, *p* = .73
BE	4.45 (.85)	.71	4.50 (.79)	.80	*t*(98) = -.26, *p* = .80

*Notes: LCE* Stereotypes related to lack of capacity for exercise, *RE* Stereotypes related to risks of exercise, *BE* Stereotypes related to benefits of exercise, *M* Mean, *SD* Standard Deviation; *t*-tests: Student’s *t*-test; α: Cronbach’s alpha

### Stage 4: convergent validity and discriminant validity

In this stage, 107 (81 men and 26 women) PLHIV (M_*age*_ = 52.51; SD = 11.42) were recruited to test convergent validity. The discriminant validity of the scale was assessed using the same sample of PLHIV and a sample of 104 (62 men and 45 women) healthy adults (M_*age*_ = 28.04; SD = 10.45).

### Convergent validity

Correlational analyses indicated that stereotypes related to lack of capacity for exercise and exercise risks among PLHIV were negatively related to exercise self-efficacy and level of physical activity. Exercise risks and lack of capacity for exercise were negatively related to patient-reported health-related quality of life. The exercise benefits stereotypes subscale was positively related to self-efficacy and level of physical activity (see Table 3). Quality of life did not reveal a significant relation to the exercise benefits subscale. Cronbach’s alphas were .77, .69 and .76 for exercise benefits, risks and lack of capacity for exercise respectively.

**Table 3:** Matrix of Pearson’s r Correlations in Stage 4 (*N* = 109)

	BE	RE	LCE	SE	PAS	QoL
BE	-					
RE	-.15*	-				
LCE	.07	.32	-			
SE	.28	-.32*	-.48*	-		
PAS	.21	-.11*	-.18*	.41**	-	
QoL	.14	-.32**	-.45**	-.32**	-.03	-

*Notes:* BE: Stereotypes related to benefits of exercise; RE: Stereotypes related to risks of exercise; LCE: Stereotypes related to lack of capacity for exercise; SE: Self-efficacy; PAS: Physical activity score; QoL: Quality of life; *p < .05; **p < .001

### Discriminant validity

ANCOVA analyses including control for level of exercise (see Table 4) revealed significant differences between PLHIV and healthy individuals for subscales related to the HIVESS. Cronbach’s alphas were all satisfactory (.77 for exercise benefits; .69 for exercise risks; and .77 for lack of capacity for exercise) in PLVIH and in healthy individuals (.71 for exercise benefits; .80 for exercise risks; and .80 for lack of capacity for exercise).

**Table 4:** Mean (SD) Differences Between PLHIV and Healthy Individuals

	PLHIV (*N =* 108)	Healthy individuals (*N =* 104)	Between-Group Difference
LCE	2.91 (1.14)	3.20 (.90)	*F*(1, 209) = 8.45, η^2^ = .04, *p* < .001
RE	2.00 (1.10)	2.52 (1.05)	*F*(1, 209) = 15.62, η^2^ = .07, *p* < .001
BE	4.51 (1.20)	4.50 (.80)	*F*(1, 209) = 2.40, η^2^ = .01, *p >* .05

*Notes: LCE* Stereotypes related to lack of capacity for exercise, *RE* Stereotypes related to risks of exercise, *BE* Stereotypes related to benefits of exercise, *SD* Standard deviation, *η* ^*2*^ Partial eta-squared

Assessments for convergent and discriminant validity, founded on the construct validity, displayed the HIVESS as being significantly related to exercise, exercise self-efficacy and quality of life in the anticipated directions. In addition the expected differences between PLHIV and healthy individuals were evidenced as regards to negative stereotypes related to exercise risks and lack of capacity for exercise.

## Discussion

Although several scales have been developed to measure psychological constructs in people living with HIV [50], the HIV Exercise Stereotype Scale is the first scale to record exercise stereotypes in this population. This scale was developed based both on literature related to psychological barriers to exercise among PLHIV [51, 52] and existing scales measuring exercise stereotypes in the elderly [19] and cancer patients [22]. The scale consists of 14 items divided into three subscales: (a) stereotypes related to exercise benefits, (b) stereotypes related to exercise risks and, (c) stereotypes related to lack of capacity for exercise. The instrument tested in this study demonstrated good structure, strong reliability and indicators of validity.

Stereotypes related to benefits of exercise were assessed by items focused on the positive effects of exercise (e.g., “Physical activity improves the morale of HIV-infected patients”). This subscale was the most highly endorsed by the HIVESS. However, we do not observe a significant positive effect of exercise benefits on exercise self-efficacy and self-reported level of physical activity. This discordance encourages questioning the gap between acknowledging the benefits of exercise and actually being physically active [52]. Nonetheless, this gap is not specific to PLHIV but is noted in the general population [51]. Some studies link the coexistence of positive views of exercise and the lack of exercise among PLHIV to: lack of motivation or lack of time [14, 15], but also to population-specific barriers such as fear of worsening the illness [12], which seems to reflect exercise risks stereotypes.

The items describing stereotypes related to the risks of exercise for PLHIV conveys the idea that exercise increases the risk of injury (e.g., “Practicing a physical activity should be avoided by HIV-infected patients because it causes injuries”) and the risk of contamination (e.g., “HIV-infected patients do not practice physical activities because they could contaminate someone during the activity”). PLHIV showed the least agreement to this subscale, whereas healthy individuals revealed higher scores for exercise risk stereotypes for PLHIV. Furthermore, exercise risks were negatively related to exercise self-efficacy, self-reported level of physical activity and patient-reported health-related quality of life. This suggests that, although PLHIV present barriers to exercise linked to exercise risk such as fear of sustaining an injury during exercise [15], there is a certain resistance to exercise risk stereotype embodiment [17] by PLHIV as already evidenced in older adults [19].

Items describing stereotypes related to lack of capacity for exercise included items pertaining to exercise self-efficacy in PLHIV, treatment side effects, as well as perceptions of physical fatigue (e.g., “HIV-infected patients do not have enough physical resources to practice a physical activity”). This type of stereotype is consistent with existing exercise barriers previously reported in the literature in terms of low levels of exercise capacity and energy [14, 15]. Stereotypes linked to lack of capacity for exercise were negatively related to exercise self-efficacy, self-reported level of physical activity and health-related quality of life. Here again, we note that healthy individuals had a slightly higher level of endorsement than PLHIV. The moderate-level agreement of this type of stereotype is consistent with findings among cancer patients [22] and older adults [19], whereby a stereotype pertaining to loss of physical capabilities was also reported. These findings provide support to the assumption that individuals are more likely to develop negative stereotypes when the latter have no strong self-referential implications [52].

The scale indicated good internal consistency and stable test-retest reliability. The correlations and regression analyses confirm convergent validity of the HIVESS with a measure of exercise self-efficacy, the level of physical activity and patient-reported health-related quality of life. The negative HIV exercise stereotypes related to the risks of exercise and to the lack of capacity for exercise were inversely related to exercise self-efficacy, level of physical activity and health-related quality of life. No significant results were observed for positive HIV exercise stereotypes evaluating the benefits of exercise warranting further investigation. Divergent validity was supported by significant differences noted between healthy individuals and PLHIV for subscales related to lack of capacity for exercise and exercise risks with healthy individuals scoring higher for both these subscales.

## Conclusion

The HIVESS is a reliable and valid tool to measure HIV exercise stereotypes in PLHIV. However, it is important to point out some limitations of the scale. The procedure reported in this paper is limited by the sociodemographic characteristics of the samples. For example, our data includes a majority of men and it would be interesting to have a more balanced gender distribution. Moreover, test-retest reliability was run in a sample of faculty students and not PLHIV. This could possibly lead to some limits in the generalizability of the scale. In addition, the HIVESS, which was first developed in French, would need to be translated in other languages in order to be used internationally.

The HIVESS allows for further investigation on psychological barriers to exercise in PLHIV. A first line of further research could try to identify the relationship between HIV exercise stereotypes and level of exercise. A second line of research could examine the moderators of HIV exercise stereotypes endorsement (such as being physically active vs. inactive and being healthy vs. HIV-positive). A third line of research could focus on the consequences of exercise stereotypes embodiment on levels of exercise and the potential role of fatigue which has been identified as prevalent complaint among PLHIV [50, 53] affecting quality of life. The French-language HIVESS is a concise multi-item scale facilitating completion and enabling extensive use both in clinical and research environments.

## Abbreviations

CFA: Confirmatory factor analysis
CFI: Comparative fit index
HIV: Human immunodeficiency virus
HIVESS: HIV exercise stereotypes scale
ICC: Intra-class correlation coefficient
PLHIV: People living with HIV
RMSEA: Root mean square error of approximation
TLI: Tucker-Lewis index
